# Biofilm dispersion: The key to biofilm eradication or opening Pandora’s box?

**DOI:** 10.1016/j.bioflm.2020.100027

**Published:** 2020-06-01

**Authors:** Jasper Wille, Tom Coenye

**Affiliations:** Laboratory of Pharmaceutical Microbiology, Ghent University, Ghent, Belgium

**Keywords:** Active biofilm dispersion, Passive dispersion, c-di-GMP, *Pseudomonas aeruginosa*

## Abstract

Biofilms are extremely difficult to eradicate due to their decreased antibiotic susceptibility. Inducing biofilm dispersion could be a potential strategy to help combat biofilm-related infections. Mechanisms of biofilm dispersion can basically be divided into two groups, i.e. active and passive dispersion. Active dispersion depends on a decrease in the intracellular c-di-GMP levels, leading to the production of enzymes that degrade the biofilm matrix and promote dispersion. In contrast, passive dispersion relies on triggers that directly release cells from the biofilm. In the present review, several active and passive dispersion strategies are discussed. In addition, the disadvantages and possible consequences of using dispersion as a treatment approach for biofilm-related infections are also reviewed.

## Biofilms: the microbial fortress

Biofilms are multicellular structures of microorganisms in which cells are encapsulated in a self-produced matrix. These biofilms can be formed on biotic and abiotic surfaces, and can also exist as non-surface-attached aggregates [[1], [2], [3], [4]]. In biofilms, microorganisms are protected from the environment and for example, the concentration of antibiotics needed to eradicate a biofilm are up to a 1000-fold higher than the concentration needed to kill planktonic cells [[5], [6], [7], [8]]. Reduced susceptibility in biofilms is due to both resistance and tolerance against antimicrobial agents and often results in treatment failure [5,9].

A potential approach to combat biofilm-related infections, is to induce biofilm dispersion, as dispersed cells and remaining biofilm cells have been shown to be more susceptible [10]. Two main mechanisms have been described for cells to ‘escape’ the biofilm [11]. Detachment typically refers to the release of individual cells or cell clusters from the surface of the biofilm and various mechanisms of biofilm detachment have been described, including abrasion (removal of cells due to collision with particles), grazing (removal due to activity of eukaryotic predators), erosion (removal due to fluid shear) and sloughing (removal of larger pieces of the biofilm by fluid shear) [11]. The term dispersion historically refers to the escape of cells from the inside of the biofilm as a regulated response to internal or external stimuli and is considered the last stage in the developmental life cycle of the biofilm [11]. However, it has more-recently become clear that the process of biofilm formation does not always follow such a fixed developmental cycle, and that not all biofilms are surface-attached [4,12,13] and the term dispersion is now more broadly used to describe the process of cells leaving the biofilm.

## Biofilm dispersion: the two mechanisms

**Fig. 1:**
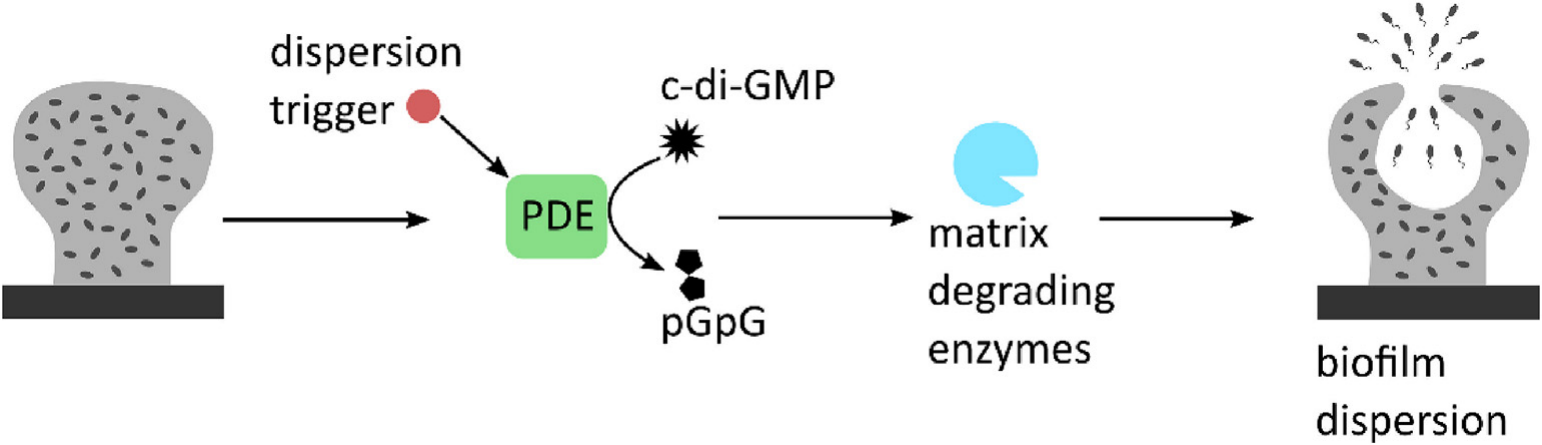
**General overview of active biofilm dispersion** During active biofilm dispersion, a dispersion trigger activates phosphodiesterases (PDEs) that hydrolyze c-di-GMP. The decreased intracellular c-di-GMP concentration leads to production of matrix degrading enzymes, resulting in dispersion.

Depending on the trigger, two forms of biofilm dispersion can be distinguished [10]. During active dispersion, the bacteria actively initiate mechanisms in response to a(n) (external) trigger, usually an environmental change, which results in the release of cells into the environment (Fig. 1) [13]. One of the mechanisms is the active degradation of the secondary messenger cyclic di-guanosine monophosphate (c-di-GMP), also called cyclic diguanylate [14]. The dispersion trigger activates phosphodiesterases (PDEs) which decrease the c-di-GMP concentration and this results in the production of matrix degrading enzymes, causing dispersal (Fig. 1) [[15], [16], [17], [18]].

Passive biofilm dispersion or biofilm detachment relies on external triggers that result in the release of single cells or clumps of biofilms [11]. Passive biofilm dispersion triggers include enzymatic degradation of the biofilm matrix and physical triggers [11,15].

### Active biofilm dispersion

#### The role of c-di-GMP

C-di-GMP was discovered as the activator of cellulose production in *Komagataeibacter xylinus* (formerly known as *Acetobacter xylinum*) [19]. The secondary messenger c-di-GMP is considered to be a universal messenger, and c-di-GMP producing and degrading enzymes have been detected in all major phyla (a list containing all known enzymes involved in production and degradation of c-di-GMP can be found on https://www.ncbi.nlm.nih.gov/Complete_Genomes/c-di-GMP.html) [18]. C-di-GMP is a major regulatory component in both biofilm development and dispersion [18]. Low intracellular c-di-GMP concentrations promote the planktonic lifestyle, while high concentrations stimulate life as a biofilm [18]. The c-di-GMP concentration is regulated by diguanylate cyclases (DGCs) and PDEs [18]. During active biofilm dispersion, the dispersion trigger leads to c-di-GMP hydrolysis by PDEs (Fig. 1) [10,15,18]. This decrease in c-di-GMP concentration activates the expression of genes involved in motility and genes involved in matrix degradation [15]. Active biofilm dispersion is induced by an environmental change. These changes can be a sudden increase or decrease in the concentration of a carbon source, an increase in the concentration of the nitrogen source, oxygen depletion, elevated levels of nitric oxide (NO), or increased heavy metal concentrations [[20], [21], [22], [23]]. Cells that disperse from the biofilm spontaneously are triggered by a lack of oxygen and nutrients in the center of the biofilm due to the matrix, which serves as a diffusion barrier [24,25]. For example, the diffusion rate of oxygen through a biofilm is only 60% of the diffusion rate through water [24] and oxygen and nutrients are actively consumed while diffusing through the biofilm, creating microenvironments [25]. In addition, oxygen is consumed by the polymorphonuclear leukocytes that attack the biofilm [26,27]. Cells near the liquid-biofilm interphase are metabolically very active due to the abundance of nutrients and oxygen, while the cells deeper in the biofilm are metabolically inactive due to reduced oxygen levels [25]. Also in biofilm aggregates an oxygen gradient is present [2]. The hypoxia is detected by sensory domains of PDEs, including PAC, PAS and H-NOX domains, which actively hydrolyze c-di-GMP [14,28,29]. The decrease in c-di-GMP concentration leads to the production and activation of matrix degrading enzymes, which results in the release of biofilm cells into the immediate surroundings [15].

Although c-di-GMP plays a major role in biofilm dispersion, lowering the c-di-GMP concentration by itself does not necessarily result in biofilm dispersion. For instance, the anti-cancer drug doxorubicin is reducing the intracellular c-di-GMP concentration in *P. aeruginosa* biofilms, while it is increasing biofilm formation by stimulating the release of extracellular DNA (eDNA) [30]. Likewise, the upregulation of PDEs does not necessarily result in biofilm dispersion [31]. For example, inducing the expression of the PDEs DipA and PA2133 does not lead to biofilm dispersion, although this induced expression does lead to lower c-di-GMP concentrations [31].

#### Nitric oxide

One of the first molecules that was identified as a biofilm dispersing agent was NO. NO is produced by macrophages in order to kill bacteria like *Mycobacterium tuberculosis* and *Salmonella* Typhimurium [32]. Although NO is toxic, it induces biofilm dispersion at low concentrations [20]. The NO-donor sodium nitroprusside (SNP) has been described to induce *Pseudomonas aeruginosa* biofilm dispersion. At a concentration of 500 ​nM, a reduction of approximately 80% of the biofilm biomass was obtained [20]. Most of the dispersion experiments have been performed with the laboratory-adapted strain *P. aeruginosa* PAO1. However, NO has also been used successfully on clinical *P. aeruginosa* isolates [33]. In order to study the role of NO in dispersal *in vivo*, *ex vivo* sputum of a cystic fibrosis (CF) patient that contained *P. aeruginosa* aggregates was used [33]. Also in these circumstances NO induced dispersal, and the mean cluster diameter of aggregates was reduced after the treatment [33].

The effect of NO on biofilms is mostly studied in *P. aeruginosa*; however, it also induces dispersion of biofilms formed by other species as well. 500 ​nM of SNP was able to reduce *Bacillus licheniformis* biofilm mass by 90%, while 10 ​μM SNP resulted in a 60% biomass reduction of a *Staphylococcus epidermidis* biofilm [34]. Besides *P. aeruginosa*, other Gram-negative bacteria such as *Escherichia coli*, *Fusobacterium nucleatum*, *Serratia marcescens* and *Vibrio cholerae* are also responsive to NO; with reductions in biofilm biomass varying from 38% for *E. coli* to 72% for *V. cholerae* [34]. NO induced dispersion is not limited to bacterial biofilms as NO application leads to a 60% reduction of a biofilm of the fungus *Candida albicans* [34]. In addition to monospecies biofilms, NO was also able to induce biofilm dispersion in a multispecies biofilm derived from a water-recycling plant, reducing the biofilm mass by 47% [34].

**Fig. 2:**
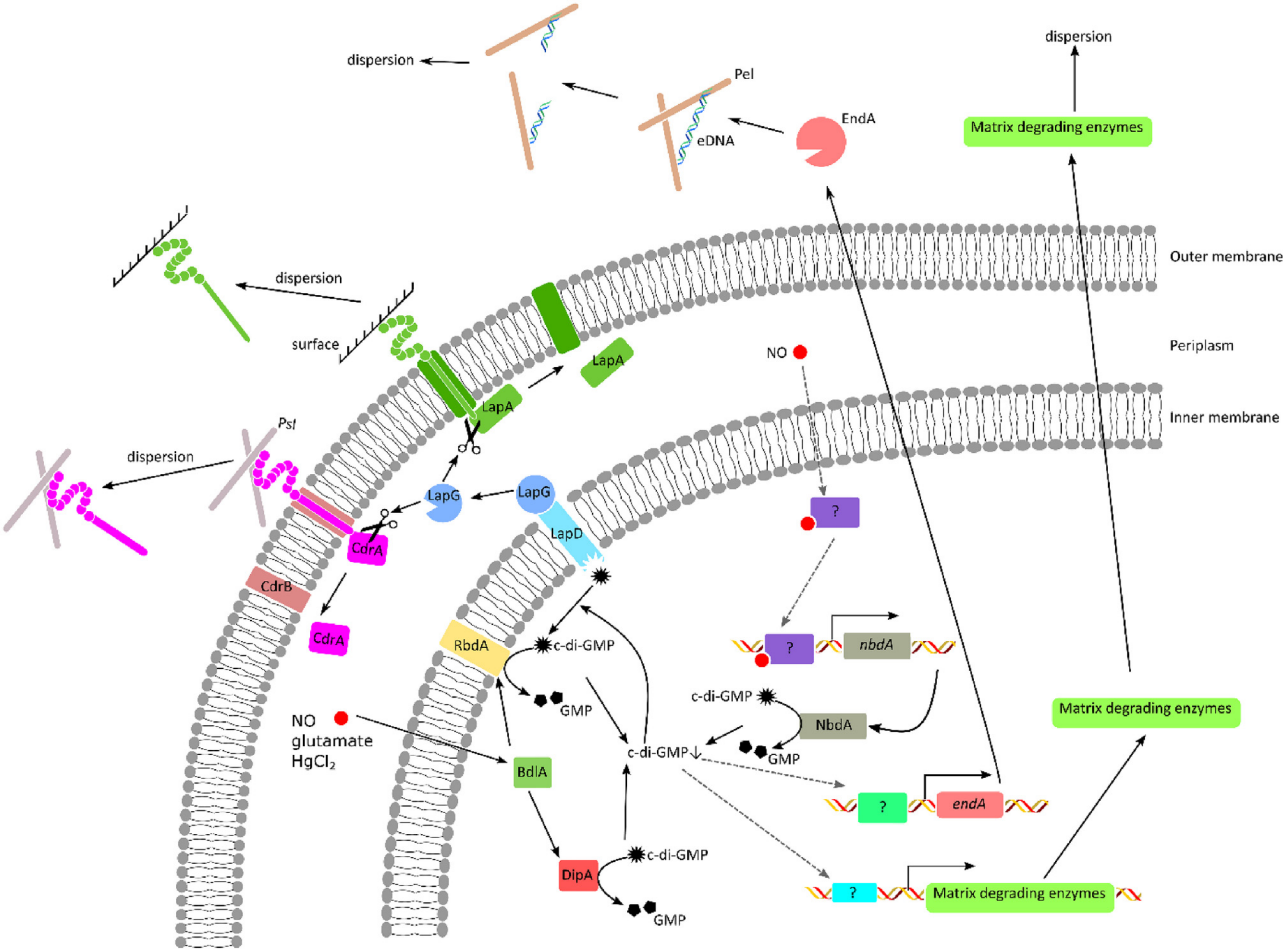
**Biofilm dispersion c-di-GMP signaling in *P. aeruginosa*** Dispersion triggers such as NO, increased glutamate concentrations and HgCl_2_ are sensed by the chemotaxis sensor BdlA. BdlA then activates the PDEs DipA and RbdA. In addition, NO increases the production of the PDE NbdA. These PDEs hydrolyze c-di-GMP resulting in a decrease of the c-di-GMP concentration. Consequently, c-di-GMP dissociates from LapD, which results in the release of the periplasmatic proteinase LapG. LapG then cleaves the Psl-bound CdrA and the surface-bound LapA, resulting in biofilm dispersion. The decrease of the c-di-GMP concentration also results in an increased production of matrix degrading enzymes such as the endonuclease EndA. The secreted matrix degrading enzymes consume the matrix components, causing cells to disperse from the biofilm. Black arrows indicate direct links, gray dashed arrows are partly unknown links.

NO activates PDEs which results in the decrease of the c-di-GMP concentration. The c-di-GMP signaling pathway in *P. aeruginosa* has been studied intensively, and in this organism NO is sensed by chemotaxis sensor BdlA, which activates the PDEs DipA and RbdA (Fig. 2) [28,35,36]. In addition, NO is sensed by an unknown sensor that activates the expression of the PDE NbdA [37]. These PDEs (DipA, RbdA and NbdA) actively hydrolyze c-di-GMP into pGpG, resulting in a decrease of the intracellular c-di-GMP concentration. Low c-di-GMP concentrations lead to a conformational change in LapD and as a consequence the periplasmic proteinase LapG is released from LapD, and degrades the matrix bound proteins LapA and CdrA [[38], [39], [40], [41], [42]]. In addition, the expression of matrix degrading enzymes is increased due to the decreased c-di-GMP concentration. These enzymes, like the endonuclease EndA, actively degrade matrix components, resulting in biofilm dispersion [43].

The biofilm remaining after NO induced dispersion is more susceptible to an antibiotic treatment than the original non-dispersed. However, it remains unclear whether this is due to an altered biofilm morphology, resulting in increased diffusion rate or due to the reduced biofilm biomass. In *P. aeruginosa*, for example, NO in combination with antimicrobial compound such as hydrogen peroxide, tobramycin or sodium dodecyl sulphate (SDS), improved the biofilm removal [20]. Furthermore, a combined exposure of aggregates in *ex vivo* sputum to NO and tobramycin, resulted in a significantly improved biofilm clearance compared to exposure to tobramycin alone [33]. The effect of combined treatment of *ex vivo* sputum with ceftazidime and tobramycin was also improved when dispersion was induced with NO [33]. A clinical trial, using NO gas, has been performed in CF patients. These patients had to inhale a mixture of air and NO gas (10 ​ppm), while antibiotics (tobramycin and ceftazidime) were administered intravenously. This resulted in a size reduction of *P. aeruginosa* clusters [33]. However, after the cessation of the test, the clusters increased to the same size as in the placebo group [33].

The combined therapy of NO and an antibiotic was not only effective against *P. aeruginosa* biofilms. NO was able of reducing a *V. cholerae* biofilm by 60%, while tetracycline reduced the biofilm biomass by 21% [34]. When tetracycline and NO were combined, the *V. cholerae* biofilm biomass was reduced by 90% [34]. NO in combination with chlorine reduced a multispecies biofilm from a waste water plant by 85–90% [34].

These data indicate that NO is a promising candidate to help eradicating biofilms both in clinical and industrial settings. However, treatment with NO in clinics, can potentially lead to systemic cytotoxicity, resulting in an increased blood pressure, pulmonary edema and even cardiac arrest [44,45]. In order to avoid this, prodrugs that release NO only at the infection site, have been developed. In contrast to NO gas and NO producing molecules (e.g. SNP), the release of NO by prodrugs is dependent on an enzymatic cleavage. For example, cephalosporin-3′-diazeniumdiolates (C3D) are cephalosporins that release NO after interactions with β-lactamases [46,47]. This was shown to actively disperse *P. aeruginosa*, non-typable *Haemophilus influenzae* and *Streptococcus pneumoniae* biofilms [46,48,49]. *P. aeruginosa* biofilms responded rapidly to C3D, resulting in a biomass reduction and an increased optical density in the effluent of a flow-cell, indicating biofilm dispersion [46]. Biofilms of *H. influenzae*, which produce β-lactamase, dispersed upon C3D administration [48]. In contrast, biofilms of *H. influenzae* without β-lactamase activity were unresponsive, indicating the importance of the β-lactamase-activity during C3D triggered biofilm dispersion [48]. However, in the case of *S. pneumoniae*, the presence of β-lactamases is not mandatory for C3D-mediated dispersal. *S. pneumoniae* contains transpeptidase/penicillin-binding proteins, which are able to cleave a C3D NO-donor (PYRRO-C3D) into an active cephalosporin and NO, which then disperses the biofilm [49]. Although C3D is a cephalosporin, the β-lactamase activity of β-lactamases like penicillinase, which is responsible for the release of NO, inactivates the cephalosporin [46,48]. Therefore the C3D induced dispersion, requires an additional antibiotic to achieve biofilm eradication. In the case of *P. aeruginosa*, a C3D treatment combined with tobramycin or ciprofloxacin, improved the biofilm clearance by at least 10-fold compared to the antibiotic treatment alone [46]. The number of *H. influenzae* biofilm cells, grown on epithelial cells, was reduced by one log during azithromycin treatment, whereas the combined therapy resulted in a 2 log reduction of the biofilm cells [48].

#### Introducing feast or famine conditions to induce biofilm dispersion

Fluctuations in nutrient concentrations have been shown to provoke biofilm dispersion. In 2004, it was demonstrated that sudden increases in the concentration of several carbon sources and a nitrogen source (NH_4_Cl) led to *P. aeruginosa* biofilm dispersal [21]. The obtained biofilm reduction varied for different carbon sources, with succinate being the most effective biofilm dispersion trigger (80% biofilm removal) [21]. Additionally, a sudden increase in the concentration of the carbon source also led to dispersal in *S. pneumoniae* and *C. albicans* biofilms [50,51]. Indeed, glucose induced dispersion in a *S. pneumoniae* biofilm that was grown on epithelial cells and *in vivo* in the nasopharynx of mice [50], while *C. albicans* biofilms could be dispersed by increasing the glucose or maltose concentration [51,52]. The c-di-GMP signaling pathway which was induced by the increase of the concentration of glutamate has been elucidated in *P. aeruginosa.* This pathway is similar to the NO induced signaling pathway (Fig. 2). The increase in the concentration of glutamate is sensed by the chemotaxis sensor BdlA, which activates the PDEs DipA and RbdA [28]. In contrast to NO-induced dispersion, *nbdA* expression is not activated during glutamate induced dispersion. Glutamate dispersion, in combination with H_2_O_2_ treatment resulted in killing of approx. 99% of *P. aeruginosa* biofilm cells, while a *bdlA* knock-out mutant (unable to disperse) remained unaffected by the H_2_O_2_ treatment [53]. In addition, the remaining biofilm after citrate or succinate dispersion had an increased susceptibility to amikacin, colistin, erythromycin and tobramycin [54].

Besides a sudden increase of nutrients, nutrient depletion also induces biofilm dispersion *in vitro*. A complete nutrient depletion results in dispersion of *Pseudomonas putida* biofilms [55]. In addition, dispersal is also observed in *P. putida* biofilms when only the carbon source is depleted [55]. Also, biofilm dispersion of *P. aeruginosa* biofilms was induced when glucose starvation was introduced. This resulted in a 60% reduction of biofilm biomass after 24 ​h of glucose depletion [56]. In *V. cholerae* biofilms, dispersal is induced when glucose or oxygen are depleted, with dispersion being more pronounced in the case of glucose depletion [57]. Also in *Staphylococcus aureus* complete glucose depletion leads to dispersion, and the remaining biofilm was more susceptible to rifampicin than a biofilm that was not dispersed [23]. Nutrient depletion can also be initiated by enzymes and might be useful in a clinical setting. For example, it was recently demonstrated that the enzyme pyruvate dehydrogenase (PDH) was able to induce *P. aeruginosa* biofilm dispersion, resulting in a 2.9-fold biofilm mass reduction [58]. In addition, biofilms of *S. aureus* biofilms also responded to a PDH treatment leading to a reduction of the biofilm biomass by 40% [58]. In contrast to NO and glutamate dispersion, PDH-induced biofilm dispersion was independent of common biofilm dispersion pathway in *P. aeruginosa* (via BdlA and the PDEs DipA and RbdA), since mutants in which these genes were knocked-out were still able to disperse [22,28,36,58]. Pyruvate depletion mediated biofilm dispersal relies on lactate dehydrogenase (LdhA) and microcolony formation regulator (MirF), since mutants in which these genes are knocked out, do not respond to PDH [58]. PDH-induced dispersion increases tobramycin-mediated killing of *P. aeruginosa* biofilms, resulting in a 5.9 log biofilm cell killing, compared to a 2.5 log killing of biofilm cells by tobramycin alone [58]. *In vivo* experiments on porcine burn wounds also demonstrated that the tobramycin susceptibility of *P. aeruginosa* was increased when the biofilm was simultaneously exposed to pyruvate dehydrogenase [58].

#### Heavy metals induce dispersion

Heavy metals also actively induce dispersion. For example, mercury chloride, silver nitrate and sodium arsenate, disperse *P. aeruginosa* PAO1 biofilms at a concentration of 2 ​mM [53]. The heavy metal induced biofilm dispersion signal pathway is the same as in dispersion by increased glutamate concentration (Fig. 2). While the applicability of some of these compounds (like mercury chloride and sodium arsenate) in healthcare is questionable due to their high toxicity [59] others like silver nitrate are currently being used (e.g. for the treatment of infected wounds) [60].

### Passive biofilm dispersion

Passive dispersion refers to the direct removal of cells from the biofilm, independent from bacterial responses such as the decrease in the c-di-GMP concentration [10,11,15]. In 1988, Breyers proposed four mechanisms of detachment that result in the release of cells from the biofilm: abrasion, shear-related removal and sloughing [61]. During abrasion, the collision of particles with the biofilm, results in the release of cells or biofilm clumps [61]. Shear-related removal is due to the continuous shear of a liquid over the biofilm which results in the erosion of single cells or aggregates from the biofilm [61]. Sloughing is the periodical release of biofilm clumps, independent from the fluid shear [61]. Finally, grazing by eukaryotic organisms like protozoa also leads to biofilm detachment [61]. Besides these natural occurring passive modes of dispersion, several techniques have been developed to induce passive dispersion. These can basically be divided into two groups: chemical (enzymatic) and physical biofilm disruption [10].

#### Enzymes consume the matrix

The biofilm matrix is composed out of three building-blocks: extracellular polysaccharides, DNA and proteins. During active dispersion, biofilm cells produce enzymes that degrade the matrix [10].

During a transposon analysis in the Gram-negative periodontal pathogen *Actinobacillus actinomycetemcomitans*, one transposon mutant was identified that showed increased biofilm thickness and failed to form satellite communities [62]. The gene which was disrupted by the transposon was *dspB*, which codes for the *N*-acetylglucosamine bond breaking enzyme dispersin B [62]. When purified dispersin B was added to the transposon mutant, the wild type phenotype was restored, indicating that dispersin B is secreted by the organism and plays an important role in dispersion of *A. actinomycetemcomitans* [62]. The exogeneous administration of dispersin B to a wild-type *A. actinomycetemcomitans* biofilm, led to an 85% reduction of the biofilm mass [62]. Characterization of the enzyme showed that it breaks the 1 ​→ ​4 glycosidic bonds of β-substituted *N*-acetylglucosamine [62]. Dispersin B also disperses *S. epidermidis* biofilms grown in various *in vitro* systems [63]. When dispersin B was used as a pretreatment against *A. actinomycetemcomitans* biofilms, the SDS susceptibility of the remaining biofilm was higher than without pretreatment [64]. A combined treatment with dispersin B and cefamandole naftate or triclosan, resulted in an improved biofilm eradication of *S. aureus* and *S. epidermidis* in comparison to the antibiotic alone [65,66]. Moreover, triclosan in combination with dispersin B also improved eradication of *E. coli* and *C. albicans* biofilms [66]. A combined treatment with dispersin B and tobramycin reduced the number of bacteria in a *S. aureus* biofilm by a 7500-fold, whereas tobramycin alone could only reduce the number of cells 40-fold [67]. Dispersin B also increased the antimicrobial activity of the peptide KSL-W against biofilms of methicillin resistant *S. aureus* (MRSA), coagulase-negative staphylococci (CoNS), *S. epidermidis*, *Acinetobacter baumannii*, *Klebsiella pneumoniae* and *P. aeruginosa* [68]. *In vivo*, dispersin B was able to eradicate a MRSA biofilm by decreasing the bioburden by 80% in combination with a silver wound dressing, whereas the silver wound dressing itself reduced the bioburden by just 14% [69].

The *P. aeruginosa* glycoside hydrolases PelA is produced when dispersion is induced [70] and. the exogenous administration of PelA and PslG induces biofilm dispersion and prevents biofilm formation. However, the dispersion capabilities of PelA and PslG are dependent on the *P. aeruginosa* biofilm composition [[71], [72], [73]]. The matrix composition of *P. aeruginosa* can be divided into four different classes, based on the extracellular polysaccharide (EPS) concentrations [73]. Class I strains form biofilms in which Pel is the dominant extracellular polysaccharide [73]. In biofilms formed by class II strains Psl is the dominant polysaccharide [73]. Strains belonging to class III and class IV are defined based upon the quantity of Pel and Psl polysaccharides in the biofilm matrix. Class III biofilm strains are redundant EPS users, since they produce relatively low amounts of Pel and Psl polysaccharides [73]. In contrast, strains in class IV are overproducing EPS [73]. Although four classes of *P. aeruginosa* strains exist based on their EPS quantity, not all strains can be divided into one class based on phenotypic characteristics [73]. PelA is more effective against class I biofilms, whereas PslG is the most efficient in removing biofilms formed by class II strains [71,72]. In addition, PslG is also more effective against biofilms of class III and class IV strains, as Psl is more abundant than Pel in their matrix [71]. Both hydrolases were able to improve the antibiotic mediated biofilm clearance of the remaining *P. aeruginosa* biofilms [71,72]. An *in vivo* experiment in mice was performed in which PslG showed synergistic anti-biofilm activity with tobramycin [72].

When DNA was discovered to be part of the biofilm matrix, it became clear that biofilms are potentially susceptible to the action of DNases such as the human (recombinant) DNase I [74] and indeed DNase I induced biofilm dispersion in*A. baumannii*, *Bordetella bronchiseptica*, *Bordetella pertussis*, *E. coli*, *Gardnerella vaginalis*, *H. influenzae*, *K. pneumoniae*, *P. aeruginosa, S. aureus*, *S. pneumoniae* and *Streptococcus pyogenes* [[74], [75], [76], [77], [78], [79]]. In *P. aeruginosa* dispersion by DNAse I appears limited to young biofilms, while mature biofilms are not affected [74]. DNase I not only induces biofilm dispersion, but it also enhances the biofilm eradication by antibiotics and biocides. DNase I enhanced the killing of *S. aureus* biofilm cells by biocides such as benzalkonium chloride, chlorhexidine and povidone iodide significantly [75] and in combination with DNase I, the antibiofilm activity of ampicillin, azithromycin, cefotaxime, levofloxacin and rifampin against *A. baumannii*, *E. coli*, *H. influenzae*, *K. pneumoniae*, *P. aeruginosa*, *S. aureus* and *S. pyogenes* biofilms was considerably increased [77,80]. DNase I has been tested in various *in vivo* models against biofilms of *S. aureus*, *B. bronchiseptica*, *B. pertussis* and *G. vaginalis* [75,78,79]. In *Caenorhabditis elegans,* the addition of DNase I in combination with tobramycin increased nematode survival following *S. aureus* infection [75]. DNase I treatment of *B. bronchiseptica* and *B. pertussis* biofilms formed on mice nasal septa reduced the biofilm drastically [78]. Finally, the biofilm of *G. vaginalis*, grown in mice, was reduced 10-fold in cell number when dispersed with DNase I [79]. One clinical trial has been performed in which DNase I was aerosolized in the lungs of CF patients, in combination with their routine antibiotic treatment and the prevalence of several pathogens (especially *S. aureus*) was drastically reduced [81].

There is also an interest in other DNases including those produced by bacteria. For example the DNase NucB produced by *B. licheniformis* EI-34-6 dispersed the biofilms of *B. licheniformis*, *Bacillus subtilis*, *E. coli*, *Micrococcus luteus* and *Pseudomonas* species, and did so more efficiently than DNase I [82].

Proteins in the biofilm matrix make the biofilm susceptible to the action of proteinases. Proteinase K dispersed a biofilm associated protein (Bap) positive *S. aureus*, while a Bap negative *S. aureus* biofilm remained unaffected [83]. In addition, the antibiotic mediated biofilm clearance increased upon proteinase K dispersion [83]. Moreover, the enzyme was also able to disperse the biofilm of *Listeria monocytogenes* within 5 ​min [84]. At concentrations higher than 1.6 ​μg/mL proteinase K, the entire biofilm was removed in 60 ​min [84]. Other proteinases such as papain and bromelain also can disperse *S. aureus* and *L. monocytogenes* biofilms [85].

#### Physical biofilm disruption

In many physiological conditions biofilms are exposed to fluid shear which erodes single cells from the biofilm surface as well as biofilm aggregates [15]. Moreover, sudden changes in the flow-rate results in dispersion. For instance, a sudden increase of the shear stress, results in an immediate release of cells from the biofilm [86]. For example, a ten-fold increase of the shear forces results in a 85% reduction of the biofilm biomass of a 67 ​h old *Streptococcus mutans* biofilm [87]. However, the influence of the shear force on biofilm dispersal is dependent on the biofilm age and the nutrient availability. An older *S. mutans* biofilm (115 ​h old) required a higher shear force than a younger *S. mutans* (67 ​h old) biofilm in order to remove 50% of the total biofilm biomass within 10 ​min [87]. In addition, the concentration of the carbon source also influences the shear-force induced biofilm dispersion [88]. For example, medium containing 1% sucrose results in a thicker *S. mutans* biofilm than medium containing 0.1% sucrose and the force needed to detach the biofilm was higher for the biofilm grown in the presence of higher sucrose concentrations [88].

Applications based on increased shear forces have been developed in order to improve the biofilm eradication. One example of such an approach that is currently used is hydro-debridement of (chronic) wounds [89,90]. During hydro-debridement, a water-jet is applied at the infection site, which removes biofilm and necrotic tissue from the wound, improving wound healing [89,90]. In dental care shear fluids are also used to remove biofilms. Dental water jets (i.e. high-pressure pulsating water) can be used to remove dental plaque; manual brushing combined with a dental water jet is up to 6 times more efficient in removing dental plaque than manual brushing alone [91]. Also in other *in vitro* and *ex vivo* studies, the value of such water jets to disperse oral biofilms was demonstrated [90,91]. During a 3 ​s treatment, the dental water jet is able to remove up to 99.99% of saliva biofilm biomass, which was grown *ex-vivo* on dental slices [92].

Other techniques, such as ultrasound, laser induced shockwaves and electrical currents, have been developed in order to passively disperse biofilms. Ultrasound induced biofilm dispersion relies on two events that disrupt the biofilm: the motion of water resulting in shear forces and the formation of cavitation bubbles [93,94]. Ultrasound treatment significantly reduced *E. coli* and *S. aureus* biofilms on stainless steel [93,94] and although ultrasound itself is not harmful to the bacteria, the ultrasound treatment alters the biofilm morphology and increases the antibiotic susceptibility [[95], [96], [97]]. *In vivo* experiments using *E. coli* biofilms, grown on polyethylene disks and on bone cement in rabbits, showed that ultrasound treatment enhanced the efficacy of gentamicin, while no bacteremia was observed [98,99]. Furthermore, an *in vivo* test using *S. epidermidis* biofilms grown on polyethylene, showed an increased susceptibility to vancomycin after ultrasound treatment in rabbits; septicemia was also in this case not observed [100]. Ultrasound is currently being used to improve the treatment of a dental root canal infections [101,102]. The eradication of a biofilm in the isthmus, a small channel between two dental roots, is extremely difficult but ultrasound was able to significantly reduce the biofilm biomass in the isthmus compared to the conventional needle irrigation technique [102].

Generating laser induced shockwaves is another approach to reduce biofilm biomass by generating liquid shear forces. Several methods to generate laser-induced shockwaves have been described. For instance, when a laser is pulsing on titanium, plasma generated shockwaves are produced [103]. These shockwaves are able to disrupt biofilms grown on different kinds of medical devices [104]. Biofilms remaining after exposure to shockwaves are more susceptible to antibiotics than biofilms not exposed to shockwaves [105]. Another method used a black polystyrene cover, which was placed over a prosthetic graft with an antibiotic solution between the cover and the graft [106]. When the laser light hits the polystyrene cover, the light energy is absorbed by the material, causing an thermal expansion resulting in a shockwave which disrupts the biofilm [106]. This shockwave was not harmful for the bacteria, but resulted in a significant decrease in *S. aureus* and *S. epidermidis* cells in combined therapy with antibiotics [106]. In order to make this method more applicable for clinical use, the transfer of laser energy to mechanical energy of different materials was determined and it was shown that polycarbonate and polyester are the best materials to generate these shockwaves [107]. When a *S. epidermidis* biofilm formed in an *ex vivo* pigskin model was treated with laser generated shockwaves and polycarbonate as the energy transferring medium, the biofilm was reduced by 52% [108]. Additionally, when the polycarbonate was coated with a titanium layer, the biofilm disrupting effect of the shockwave was improved and resulted in a biofilm reduction of 80%; in addition the anti-biofilm effect of gentamicin was potentiated by these laser generated shockwaves [109,110]. Laser induced shockwaves are currently also used in dental care, where they are induced by the rapid energy absorption which creates water vapor bubbles that upon implosion disrupt the biofilm [102,111,112].

**Fig. 3:**
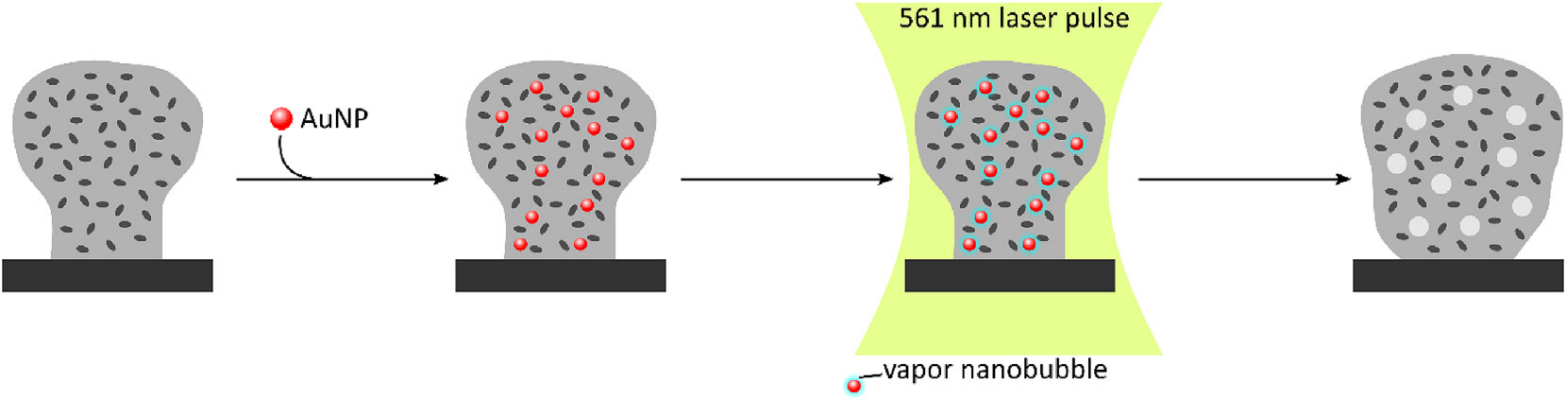
**Schematic overview of VNB-formation** Prior to VNB formation, 70 ​nm AuNPs are added to the biofilm, which will penetrate through biofilm. Subsequently, the biofilm is submitted to a 561 ​nm laser pulse (7 ns), which is heating up the AuNPs rapidly leading to the formation of water vapor bubbles or VNBs around the AuNP. The expansion of the VNBs is, followed by the implosion of the bubble, altering the biofilm density and biofilm morphology.

Similarly, laser induced vapor nanobubbles (VNB) can be used to disrupt biofilms. In order to induce VNBs in a biofilm, gold nanoparticles (AuNPs) are added and after penetration through the biofilm they are excited with a green pulsing laser (Fig. 3) [113]. The heat generated in the excited AuNPs is subsequently transferred to the surrounding water, that evaporates, leading to the formation of VNBs. The expansion of the water vapor, followed by the implosion of the bubble, generates small fluidic frictions that disrupt the biofilm [113]. VNB treated *Burkholderia multivorans*, *P. aeruginosa* or *S. aureus* biofilms showed an increased susceptibility to antibiotics compared to an untreated biofilm and increasing the number of laser pulses leads to the formation of multiple VNBs, which increased the antibiotic susceptibility of the remaining biofilm [113]. Besides increasing the antibiotic susceptibility, VNBs also improve the biofilm eradication by disinfectants [114]. *P. aeruginosa* biofilms were more susceptible to benzalkonium chloride after VNB treatment, while for *S. aureus* biofilm eradication by cetrimide or mupirocin was improved [114].

Another method to passively disrupt biofilms is by applying a low electrical current which causes the biofilm to detach from the surface [[115], [116], [117]]. The application of an electric current on stainless steel studs resulted in a 10-fold reduction in the number of *P. aeruginosa* biofilm cells [115]. The electric current was also able to induce *S. epidermidis* biofilm dispersion from stainless steel [116,117]. Due to the electrolysis of water, hydrogen gas is produced at the cathode, while oxygen gas is produced at the anode and the formation of these gas bubbles disrupts the biofilm [117]. The remaining biofilm cells are more susceptible to biocides and antibiotics and in fact, during the exposure to the electric current, the antibiotic efficacy is increased due to the bioelectric effect [[118], [119], [120]]. This bioelectric effect is not fully understood yet, although several factors potentially contributing to the increased efficacy have been described. The oxygen and reactive oxygen species (ROS) that are produced duringthe electrolysis of water contribute to the increased susceptibility [121,122]. In addition, the electric current reduces the antibiotic binding capabilities of the matrix and increases permeability of biofilm cells (electroporation), leading to an increased antibiotic uptake in the cells [122]. The effect of a low electric current has also been evaluated *in vivo* using rabbits [123]. A spinal coupling device containing 10^6^ ​CFU *S. aureus* was implanted, and the electric current in the implant was generated by introducing a magnetic field over the skin of the animal. In combination with systemically administered ceftriaxone this led to a significant reduction in the number of bacteria on the implant, while there was no difference in microbial load in the surrounding tissue, indicating that the electric current only induces biofilm dispersion on the device in which the electric current is applied [123].

## Biofilm dispersal: opening Pandora’s box?

Both active and passive biofilm dispersion result in a reduction of biofilm biomass and the remaining biofilm cells are more susceptible to antibiotics than cells in an undispersed biofilm. However, it remains unclear whether this increased antibiotic susceptibility is due to a loss of biofilm biomass or a modification of the biofilm morphology, allowing a better penetration of antimicrobial compounds through the biofilm. While biofilm dispersion is considered a potentially useful strategy to improve antibiotic treatment of biofilm infections both active and passive dispersion methods have disadvantages. Although it has been suggested that dispersed cells return to the bulk as planktonic cells, it was shown in 2014 that dispersed *P. aeruginosa* cells are distinct from biofilm cells and from planktonic cells [124]. For example, the transcriptome of dispersed cells differs from that of both biofilm and planktonic cells [124]. In addition, dispersed cells have lower c-di-GMP concentrations in comparison to planktonic cells. Lower c-di-GMP concentrations have been linked with increased virulence, suggesting that dispersed cells are more virulent compared to planktonic cells [125]. Indeed, virulence assays confirmed that dispersed cells were more effective in penetrating and killing macrophages, than their planktonic counterparts [124]. Moreover, the dispersed cells appeared to be more effective in killing *C. elegans* than planktonic cells [124]. These results indicate that the dispersed cells, which have the potential to disseminate through the body, can worsen the clinical outcome. Indeed, *in vivo* studies have described a spreading of the infection after dispersal events [50,126]. While nasopharyngeal colonization of *S. pneumoniae* is common and is usually asymptomatic [127], a secondary infection with an influenza A virus leads to biofilm dispersal of *S. pneumoniae* [50]. This results in a colonization of the lung, causing pneumonia, and colonization of the middle ear, leading to acute otitis media [50]. Moreover, these dispersed cells indicated to be hypervirulent, killing the mice via bacteremia [50]. Also *S. aureus* can asymptomatically colonize the nasopharynx [128] and *in vitro* and *in vivo* assays have demonstrated that *S. aureus* biofilm dispersion was also induced upon an influenza A virus infection of the epithelium cells [129]. Interestingly, when *S. aureus* and *S. pneumoniae* were cocultured on epithelial cells, a secondary influenza A infection was able to induce dispersion of *S. pneumoniae*, but not of *S. aureus*, clearly indicating that induction and regulation of dispersion are complex [130].

A recent study in mice using *P. aeruginosa* and S*. aureus* biofilms showed that in infected wounds induction of dispersion by glycoside hydrolases leads to a fatal septicemia and bacteria could be detected in the blood of the mice within 5 ​h after the biofilm dispersal trigger was given [126]. However, the administration of meropenem, both systemic and topical in combination with the dispersion trigger, prevented the septicemia and resulted eventually in biofilm eradication and wound healing [126]. While meropenem was able to prevent bacteremia in this study, the antibiotic susceptibility of the dispersed cells depends on the biofilm dispersion trigger [31]. *P. aeruginosa* cells that were dispersed by a sudden increase of the concentration of NO had a similar susceptibility to tobramycin and colistin as planktonic cells [31]. However, cells that were dispersed by an increased glutamate concentration were less susceptible to colistin than SNP dispersed cells and planktonic cells [31]. This indicates that successful prevention of septicemia by the use of antibiotics, will dependent on both the biofilm dispersion trigger and the antibiotic.

There are other questions that need to be addressed before active and/or passive dispersion can be applied in the treatment of biofilm infections, including timing and concentration. For example, during active biofilm dispersion, the actual moment when cells detach from the biofilm is 10–15 ​min after the biofilm dispersion trigger is given [31]. In addition, concentrations of the active biofilm dispersion trigger below a certain threshold can actually prevent biofilm dispersion by the bacteria. E.g. low NO concentrations lead to the production of flavohemoglobins by *P. aeruginosa* and when the NO concentrations are subsequently increased, the NO is scavenged by these flavohemoglobins, preventing biofilm dispersion [131]. In contrast to active dispersion, passive dispersion triggers only require short contact times (up to 3 ​min) to significantly reduce the biofilm biomass but these passive dispersion triggers can also cause more harm to the surrounding tissue [98,[132], [133], [134], [135]]. However, *in vivo* studies in which passive biofilm dispersion was used have so far not reported any form of septicemia [[98], [99], [100]].

## Concluding remarks

Is biofilm dispersion the key to biofilm eradication or is it opening Pandora’s box? Both active and passive biofilm dispersion are promising approaches as they reduce the biofilm biomass and increase the susceptibility of the remaining biofilm cells. However, dispersed biofilm cells are still not well studied, although that they potentially can cause bacteremia [126]. It is anticipated that these dispersed biofilm cells can be prevented from causing septicemia by the use of antibiotics [126] although it should be noted that the nature of the biofilm dispersion trigger plays a role in determining the antibiotic susceptibility of the dispersed cells [31]. Combined the available data suggest that care should be taken to use biofilm dispersal as part of anti-biofilm strategies and that data about susceptibility of the dispersed cells is required before this approach is introduced in clinical practice.

## Declaration of competing interest

None.
